# Stillbirths among Pregnant Women Admitted to the Department of Obstetrics and Gynaecology in a Tertiary Care Centre: A Descriptive Cross-sectional Study

**DOI:** 10.31729/jnma.7758

**Published:** 2022-09-30

**Authors:** Damber Khadka, Keshar Bahadur Dhakal, Astha Dhakal, Sulochana Dhakal Rai

**Affiliations:** 1 Department of Obstetrics and Gynaecology, Karnali Province Hospital, Birendranagar, Surkhet, Nepal; 2 Faculty of Health and Social Sciences, Bournemouth University, St. Pauuls Lane, Bournemouth, United Kingdom

**Keywords:** *antenatal screening*, *birth weight*, *stillbirth*

## Abstract

**Introduction:**

Stillbirth is often defined as the death of a foetus in the uterus prior to its birth or during the process of birth. Most of the stillbirths are preventable global health problem. The aim of this study was to find out the prevalence of stillbirths among pregnant women admitted to the Department of Obstetrics and Gynaecology in a tertiary care centre.

**Methods:**

A descriptive cross-sectional study was conducted in the Department of Obstetrics and Gynaecology in a tertiary care centre among pregnant women admitted between 14 April 2021 to 13 April 2022. Ethical approval was taken from the Institutional Review Committee (Reference number: 43). Convenience sampling method was used. The data were collected from the medical record section using a proforma. Point estimate and 95% Confidence Interval were calculated.

**Results:**

Among 5,118 pregnant women, stillbirths were found in 126 (2.46%) (2.04-2.88, 95% Confidence Interval).

**Conclusions:**

The prevalence of stillbirth among pregnant women was higher than in the other studies done in similar settings.

## INTRODUCTION

Stillbirth is defined as the death of a foetus weighing 500 grams or more; or if birth weight is unknown, by gestational age of 22 weeks or more.^1^ Globally, one stillbirth occurs every 16 seconds and about 2 million stillbirths occur each year.^2,3^ The national rate of stillbirth was reported to be 18.0 per 1000 live births in 2015.^4^ There exist pronounced spatial variations in stillbirth in Nepal, and the rate is as high as 35 per 1000 births in a rural district of Nepal.^4,5^

The incidence significantly varied across provinces with the highest stillbirth rate in Madhesh province.^5^ Despite the vast majority of stillbirths occurring in Nepal, as to our search of the literature, there is no study done on the pooled prevalence of stillbirths in Karnali province.

This study aimed to find out the prevalence of stillbirth among pregnant women admitted to the Department of Obstetrics and Gynaecology in a tertiary care centre.

## METHODS

A descriptive cross-sectional study was conducted among pregnant women admitted to the ward of the Department of Obstetrics and Gynaecology of the the Karnali Province Hospital among pregnant women admitted between 14 April 2021 to 13 April 2022. Ethical approval was taken from the Institutional Review Committee (Reference number: 43). Pregnant women visiting the department during the study period were included in the study whereas missing data were exlcuded from the study. A total number of deliveries was obtained from the medical record section (MRS) and maternity register on monthly basis. Convenience sampling was used. The sample size was calculated by using the following formula:


$$\begin{array}{ll} \text{n} & ={\text{Z}}^{2}\times \frac{\text{p}\times \text{q}}{{\text{e}}^{\text{2}}}\\ & =1.96^{2}\times \frac{0.5\times 0.5}{0.02^{2}}\\ & =2401 \end{array}$$

Where,

n= minimum required sample size
Z= 1.96 at 95% Confidence Interval (CI)
p= prevalence taken as 50% for maximum sample size calculation
q= 1-p
e= margin of error, 2%

The minimum required sample size was 2,401. Doubling the sample size, the sample size was 4,802. However, a sample size of 5118 was taken. Stillbirths were diagnosed with intrauterine fetal death (IUFD) at or after 28 weeks gestation, occurring during the process of delivery or during the intrapartum period.^3^ At first, a list of stillbirths was obtained from the MRS during the study period. Next, a list of all patients registered as stillbirth in the maternity register was obtained for the same period. Both lists were then verified and compared together to eliminate the chances of repetition or missing the cases. All in-patient files as per the list were collected from the obstetric ward and MRS. Finally, the required data were extracted from these files, according to the proforma developed prior to data collection. Information on possible causes of stillbirths was also obtained from the documented conclusions of monthly perinatal review meetings.

Information (related to stillbirths) on maternal demographic characters (age, address, occupation and education) was obtained. Relevant obstetrical factors (parity, gestational age, ANC visits and presenting complaints) and risk factors related to stillbirth were also collected. Furthermore, frequently observed risk factors for stillbirths were also obtained. Foetal characteristics related to stillbirth were also studied and the information on birth weight, foetal maturity, type of stillbirth, the coexistence of congenital anomalies, and possible causes of stillbirth were obtained from the patient files.

The collected data were entered and analysed in Microsoft Excel 2017. Point estimate and 95% CI were calculated.

## RESULTS

Among 5,118 pregnant women, stillbirth was found in 126 (2.46%) (2.04-2.88, 95% CI). Seventy four (58.80%) were from Surkhet district followed by 20 (15.90%) cases from Dailekh district and 32 (25.40%) cases were from other parts of the province. In this study, 75 (59.50%) stillbirths belonged to Brahmin/ Chhetri ethnicity followed by 50 (39.70%) cases from Janajati. About 92 (73%) mothers were Hindu followed by eight (6.34%) Buddhists and one (0.79%) Muslim.

**Figure 1:**
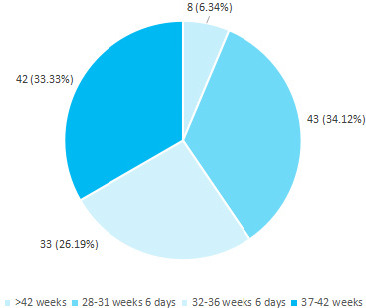
Distribution of stillbirth according to weeks of gestation (n= 126).

About 76 (60.31%) of stillbirths were preterm (before 37 weeks of gestation). Among them, 33 (26.19%) cases were observed prior to 32 weeks. Similarly, 42 (33.33%) stillborn babies were delivered full term (37-42 weeks). In this study, eight (6.34%) cases of stillbirth occurred after 42 weeks (post-term) of gestation (Figure 1).

**Figure 2:**
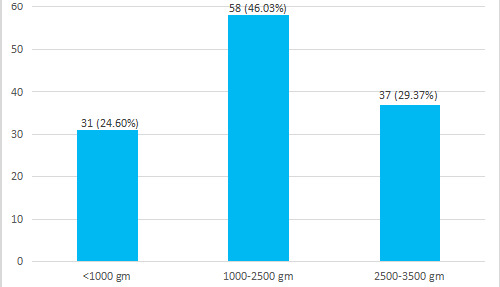
Distribution of weight of stillborn babies (n= 126).

Among 126 cases of stillbirths, foetal heart sound was absent in 103 (81.70%) cases, however, foetal heart sound (FHS) was recorded in 23 (18.20%) at the time of admission. Most of the stillborn babies 112 (88.90%) were delivered with normal vaginal delivery followed by caesarean section in 11 (8.70%). Three (2.38%) cases were born with vacuum delivery. The majority, 58 (46%) of the stillborn babies had their birth weight between 1000-2500 grams (Figure 2).

Among stillbirths babies, females were 85 (67.46%) and 41 (32.53%) were males. Also, 14 (11.10%) had some forms of congenital anomalies identified at the time of birth. Macerated babies were 103 (81.70%) and fresh stillbirths were 23 (18.20%) at the time of delivery.

A total of 106 (84.10%) who had a stillborn baby were required to stay in hospital for 3 days or less. However, 20 (15.90%) mothers needed to be admitted to the hospital for more than 3 days. Stillbirth was present in 53 (42.06%) in primiparous women (Table 1).

**Table 1:** Parity-wise distribution of stillbirth (n= 126).

Parity	n (%)
Primiparity	53 (42.06)
2-3	38 (30.15)
4-5	30 (23.80)
6 or more	5 (3.96)

In this study, around two-thirds, 84 (66.70%) women visited ANC (antenatal check-up) clinics at least one or more times however, the number of ANC visits varied individually among the mothers. The majority of mothers, 72 (57.10%) were presented with loss of foetal movements and about one-third of mothers, 42 (33.30%) were presented with no clinical presentations. Nine (7.10%) women were presented with other complaints. APH was the presenting complaint in three (2.40%) cases (Table 2).

**Table 2:** Co-morbidities among pregnant women (n= 126).

Co-morbidities	n (%)
Hypertensive disorders	8 (6.30)
Maternal diabetes/chronic illness	13 (10.30)
Malpresentation	21 (16.70)
Maternal UTI and other infections	7 (5.50)
Not identified risk factors	77 (61)

**Figure 3:**
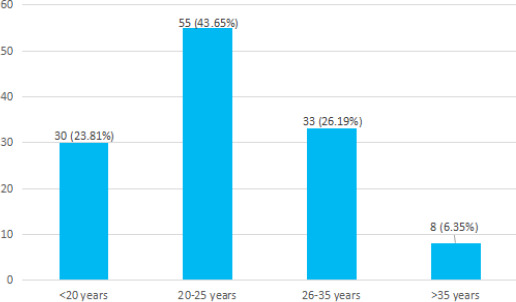
Distribution of stillbirth and maternal age (n= 126).

A total of 55 (43.65%) belonged to the age between 20 to 25 years followed by 33 (26.19%) in the age group 26 to 35 years (Figure 3).

Most mothers with stillbirths were housewives 121 (96.03%). Five (9.00%) mothers were involved in service/office work or business apart from agriculture. A total of 22 (17.50%) mothers were illiterate. The majority of mothers had secondary level education of 6 or more years of schooling and a small number of mothers had primary education of 5 or fewer years of school education. Seven (5.60%) were educated at the university level.

## DISCUSSION

The prevalence of stillbirths in Karnali Province Hospital is found to be high (24.61 per 1000 total births). The national stillbirth rates across the world varied between 1.4 to 32.2 per 1000 total births in 2019.^2^ The incidence was found to be 15 and 11.2 per 1000 births studied conducted in Nepal.^6-9^ Similarly, the incidence of stillbirth was 16 per 1000 births in a study conducted in Sudan.^10^ The incidence of IUFD in Nepal was observed as low as 8 per 1000 births in one study.^11^ However, the incidence was found to be high as 39 per 1000 births in another study in India.^12^ The incidence of stillbirth in our study is comparable to the incidence of 19.6, 23.5 and 22 per 1000 births observed in some other studies.^13-15^ Compared to available studies on stillbirth, the incidence of stillbirth found in this study is possibly the highest compared to other South Asian countries.^16^

It is found that stillbirth is more common among mothers aged 20'25 years (43.65%) in this study. Nearly 70% of stillbirths occurred among mothers aged between 20'35 years. Stillbirths among teenaged mothers are found notable, comprising 23.80%. It is comparable to the study carried out in India in which 32.1% of stillbirths occurred in mothers of age 21-25 years and 33.9% of stillbirths were found in mothers aged 16-20 years.^12,14^ Similarly, 82.9% of stillbirths were found in mothers between 20-34 years in another study conducted in Nepal. It is detected that most stillbirths occur in the age group 20-35 years.^15^

In our study, mothers with primiparity constituted 42% of all stillbirths. This result is comparable to the few other studies.^6-8^ Additionally, 57% and 50.7% of stillbirths were observed in primiparous mothers in some studies.^16-19^ The percentage of primiparous mothers with stillbirths in the present study is very close to some other studies.^19-21^

In our study, 60.3% of stillborn babies were preterm. Among them, 26.2% were born before 32 weeks of gestation. Similar findings of 'preterm stillbirths' were observed also in a few other studies.^12-16^ Likewise, more than half of all stillbirths were found in preterm births in some studies.^18-21^ In this study, 57.1% of mothers with stillbirth were presented with loss of foetal movements. However, 33.3% of mothers presented with no clinical symptoms but were diagnosed as IUFD on routine ultrasound scanning. Only 2.4% of mothers were presented with APH on admission. Similarly, 77.1% of mothers were presented with reduced foetal movements.

In this study, few risk factors were found in 38.9% of cases of stillbirths. However, in most cases, there were no identified risk factors present. Malpresentation was the most frequently observed risk factor for stillbirths observed only 16.7% followed by maternal diabetes and other chronic illnesses (10.3%). Unexplained/ unidentified causes of death in stillbirths were high (65.9%) in this study. However, unknown/unexplained cause of death in stillbirths is reported to be lower than our study in other studies. Hypertensive disorders in pregnancy were the most common.^7,9,21^ In our study, most of the stillborn babies (88.9%) were delivered with normal vaginal delivery. Vaginal delivery was the main mode of delivery also in other studies (94.9%, 77.5% and 86.2% and 94.9%).^6^

Our hospital still operates on a paper-based recording system, so that, it was very challenging to access data. This study being conducted in a single centre of a Western Nepal, the findings might not be generalizable to other populations. So, the nationwide epidemiological study should be conducted to find out the prevalence of stillbirths along with causative factors among deliveries for which higher study designs are recommended.

## CONCLUSIONS

The prevalence of stillbirth among pregnant women was higher than in the other studies done in similar settings. Hence, the provision of quality maternity care, early diagnosis and availability of evidence-based interventions is vital to minimise stillbirth.
